# The Story of the Insane from Year to Year

**Published:** 1901-07-20

**Authors:** 


### THE STORY OF THE INSANE FROM YEAR TO YEAR.

(THIRTEENTH SERIES.)

### LONDON COUNTY ASYLUM AT CANE HILL.

THE total number of patients on January 1st, 1899, was 2,218, and on December 31st it was 2,202. There were 488 first admissions, 125 re-admissions, 166 recoveries, 88 discharged relieved, 198 discharged not improved, and 177 deaths. The average number resident during the year was 2,211, and the total number of persons under care was 2,831. Excluding transfers from other asylums, and calculating the recoveries on the ordinary admissions, the recovery rate stands at 35·85 per cent. Calculated on the average number resident the deaths stand at 8 per cent. Of the year's deaths 80 were caused by cerebral and spinal diseases, 28 by consumption, 8 by pneumonia, 7 by bronchitis, 2 by congestion of lung, and 1 by colitis. Of the year's admissions, the insanity was caused by domestic trouble in 61, by adverse circumstances in 52, by religion in 16, by love in 9, by intemperance in drink in 87, and by hereditary influence 72. In 128 cases the cause of the insanity is stated to be "previous attacks," and unquestionably many of these 128 ought really to be in one of the two last-named causes. In 82 cases no cause was known. The deaths from phthisis were nearly 16 per cent. of the total number of deaths; but Dr. Moody does not give us his views on this question of the treatment of phthisis which is vexing the souls of so many medical superintendents. The death rate from other lung diseases was, however, not high. The fatal case of colitis is not referred to. Dr. Moody was severely wounded by a patient, and had to be absent from his duties for six months. We are pleased to learn that much benefit resulted from his prolonged rest. The Committee express their appreciation "of the manner in which Dr. Moody, well aided by the assistant medical officers and the asylum staff generally, has performed his duties." The weekly cost of maintenance per head was a fraction over 10s.

### THE WEST RIDING ASYLUM AT MENSTON.

ON January 1st, 1899, the Menston Asylum contained 1,530 patients, and on December 31st this number had risen to 1,562. There were 492 first admissions, 72 re-admissions, 273 recoveries, 28 discharged relieved, and 189 deaths. The average daily number resident was 1,587, and the total number of persons under treatment during the year was 2,081. The percentage of recoveries is very high. It stands at 50·83, calculated on the admissions, and the deaths, also high, stand at 11·90 per cent., calculated on the average number resident. Of the deaths which occurred during the year, 78 were caused by cerebral and spinal diseases, 24 by consumption, 4 by pneumonia, 6 by bronchitis, 5 by other lung diseases, 2 by enteritis, and 1 by enteric fever. Table X. shows that in 219 of the year's admissions such moral causes as domestic troubles, mental anxiety, and religion had something to do with the cause of the insanity; in 73 drink was the assigned cause, and in 135 hereditary influences. As already stated, there were two deaths from enteritis, and there would seem to have been about ten cases of that disease, but Dr. McDowall does not state his views on this asylum scourge which so often defies all attempts to permanently dislodge it. Dr McDowall has four assistant medical officers and three clinical clerks (one of the latter being a lady and also one of the assistants) to help him in his work. The lady assistant medical officer has £130 a year as salary, and we can hardly imagine a better career for a lady doctor than asylum work, nor a better or more useful officer for the asylum, but whether the salary here given is sufficient to retain the services of such an officer is extremely doubtful, because she cannot be imbued with the hope of one day filling the chief office, and it is this cheering ray of hope that keeps so many assistant medical officers from wandering. During the year fourteen of the staff obtained the nursing certificate of the Medico-Psychological Association. The Commissioners in Lunacy "cannot refrain from again referring to the absence of a chapel," but neither the Committee of Visitors nor the medical superintendent seem to desire this adjunct, at least they do not refer to it in their reports, and if the new chapel were to be a detached building their present plan of using the hall is infinitely preferable. The committee "beg to put on record their appreciation of the constant care shown by the medical superintendent and his assistants." The weekly charge for union patients is 9s. 4d. per head, while private patients pay from 10s. 6d. to 20s.

